# Labor Off-Farm Employment and Cropland Abandonment in Rural China: Spatial Distribution and Empirical Analysis

**DOI:** 10.3390/ijerph15091808

**Published:** 2018-08-22

**Authors:** Xin Deng, Dingde Xu, Yanbin Qi, Miao Zeng

**Affiliations:** 1 Collage of Economics, Sichuan Agricultural University, Chengdu 611130, China; xindeng66@126.com; 2 Sichuan Center for Rural Development Research, College of Management of Sichuan Agricultural University, Chengdu 611130, China; dingdexu@126.com; 3 School of Economics of Sichuan University, Chengdu 610065, China; zengmiao61@126.com

**Keywords:** cropland abandonment, labor off-farm employment, spatial distribution, rural China

## Abstract

Alleviating cropland misallocation is helpful for the sustainable development of agriculture. Does off-farm employment inevitably result in cropland misallocation (e.g., cropland abandonment) and threaten the sustainable development of agriculture? This study differs from prior studies in its view that off-farm employment does not necessarily result in cropland abandonment. Specifically, the study employs survey data from 8031 peasant households from 27 provinces in rural China and spatial statistics to analyze the distribution of off-farm employment and cropland abandonment. Empirical models (i.e., IV-Probit and IV-Tobit) are used to examine the quantitative relation between off-farm employment and cropland abandonment. The results are as follows. (1) The spatial distribution of off-farm employment or cropland abandonment differs among regions. Regions with a higher rate of off-farm employment show more cropland abandonment but a lower average area of cropland abandonment. (2) Off-farm employment has a significant and positive correlation with cropland abandonment. However, its square has a significant and negative correlation with cropland abandonment; i.e., there is an inverted U-shaped relationship between off-farm employment and cropland abandonment, with the turning point occurring at 46.00% off-farm employment. (3) Off-farm employment has a significant and positive correlation with the area of cropland abandonment. However, its square has a significant and negative correlation with the area; i.e., there is an inverted U-shaped relationship between off-farm employment and area, with the turning point occurring at 44.50% off-farm employment. This study reveals the relationship between off-farm employment and cropland abandonment and provides policymakers with references for use in developing sustainable agriculture.

## 1. Introduction

Factor misallocation harms the sustainable development of the economy by decreasing total factor productivity [1,2]. In agriculture, cropland misallocation may decrease agricultural productivity. For example, one study found that a lack of cropland mobility resulted in misallocation, which decreased agricultural productivity by 17% [3]. Obviously, the effective allocation of cropland is essential for sustainable agricultural development [4]. Therefore, ascertaining the determinants of cropland allocation has been a topic of substantial interest in agricultural economics and geography. A number of studies have investigated the impact of rural off-farm employment on land allocation [5,6,7,8,9,10,11,12,13]. The key idea of these studies is that off-farm employment can enhance land transfers, where land is transferred from farmers with lower productivity to farmers with higher productivity [14]. Such a development is not expected to occur in rural China, where there is a higher share of off-farm employment and a lower share of cropland transfer.

**Figure 1:**
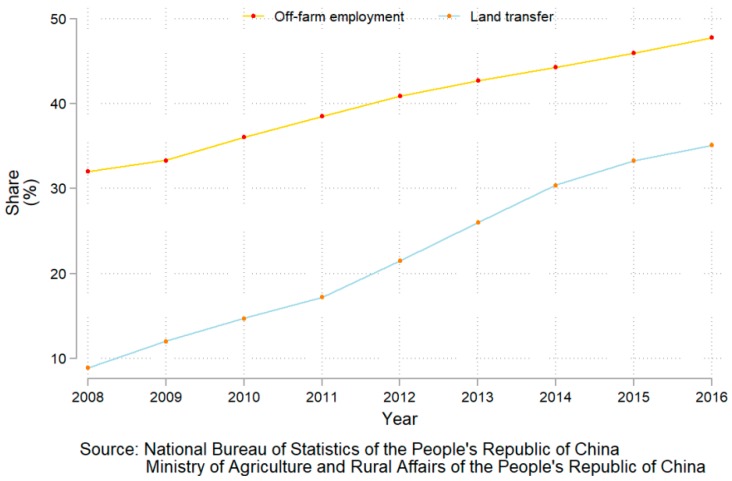
Share of off-farm employment and land transfer.

For a long time, the proportion of off-farm employment (i.e., the share of the rural off-farm employment population of the total rural population) has been higher than the land transfer proportion (i.e., the share of rural land transferred out of total rural land). As shown in Figure 1, between 2008 and 2016, the average proportion of off-farm employment was 40.18%, while the average proportion of land transfer was only 20.43%. On average, the share of off-farm employment approached twice that of land transfer. China’s rural areas generally have a higher share of off-farm employment and a lower share of cropland transfer. However, prior studies have poorly explained this phenomenon in rural China. In fact, the ways in which peasant-owned land is utilized not only include self-management and transfer but also abandonment. For example, the proportion of abandoned cropland was 13.5% in 2011 and 15% in 2013 [15], and the median of the reported ratios for the eastern, central, and western regions was 5.62%, 5.7%, and 4%, respectively [16]. Thus, researchers have gradually begun to investigate the relationship between off-farm employment and cropland abandonment [17,18,19,20,21,22].

Cropland abandonment means that the utilization frequency of cropland is decreased [23]. Abandonment occurs due to changes in social and economic factors [24], and one of the most important factors is the migration of peasants. Aide and Grau argue that the rapid development of off-farm industry resulted in cropland abandonment in Latin America [17]. Van Doorn and Bakker consider one reason for cropland abandonment in southeast Portugal to be the out-migration of peasants [18]. Other European countries also furnish evidence that off-farm employment increases cropland abandonment, for example, in the Swiss mountains [25], eastern Albania [19], and Slovakia [21]. These studies focus on Latin American and European countries to investigate the relationship between off-farm employment and cropland abandonment, and all of these countries will continue to receive scholarly attention in future research. However, as the world’s largest developing country, China has been feeding 20% of the world’s population on the basis of approximately 7% of the world’s total cropland [26]. Thus, clarifying the relationship between off-farm employment and cropland abandonment in rural China has important implications in terms of world food security.

Additionally, the spatial distribution of cropland abandonment is in disequilibrium. For instance, in the USA, regions of cropland abandonment are concentrated in the east [27,28]. However, in Europe, the central European mountainous area, the Mediterranean region, and eastern Europe are the most obvious areas of cropland abandonment [23,29,30,31]. China has a land area of 960 km^2^ (ranking third worldwide), of which mountainous areas account for approximately 70% [11,32,33,34,35]. What is the spatial distribution of off-farm employment and cropland abandonment in China’s provinces? To answer this question, it is urgent to analyze the spatial distribution of off-farm employment and cropland abandonment from a geographical perspective.

As mentioned above, it is worth clarifying the relationship between off-farm employment and cropland abandonment in rural China. In 2017, the Communist Party of China proposed the “Village Revitalization Strategy”. One core goal of the strategy is to open up channels for the bidirectional flow of talent, land, and funds between urban and rural areas [34,36]. However, in rural China, many croplands are abandoned, which not only causes serious waste of resources but also hinders implementation of the “Village Revitalization Strategy”. Therefore, the Chinese government is accelerating the work of rural land reform and guiding talent and funds back to the countryside in an effort to resolve the problem of cropland abandonment in rural China. In this context, the results of this study may provide a reference for the rational allocation of talent and land resources in urban and rural areas.

Additionally, prior studies have established a rich theoretical foundation on which to discuss the relationship between off-farm employment and cropland abandonment. However, it is a mistake to assume that off-farm employment promotes cropland abandonment, and in fact there may be a non-linear relationship between the two variables. In addition, China is the most populous country in the world; thus, clarifying the relationship between off-farm employment and cropland abandonment in rural China has important implications for efforts to increase world food security. Additionally, China’s landforms are complex, and it is crucial to analyze the spatial distribution of off-farm employment and cropland abandonment from a geographical perspective. The key scientific questions to be answered in this study are as follows:

(1) From a spatial perspective, what is the distribution of off-farm labor and cropland abandonment in rural China?
(2) From a quantitative perspective, does off-farm employment inevitably result in cropland abandonment?

## 2. Theoretical Analyses

The new economics of labor migration theory indicate that off-farm employment is undertaken to maximize household income [37] and, more importantly, to spread family risk [38], the essence of which is the reallocation of household labor resources [39]. This study considers that a rational peasant driven by economic interests would allocate household labor resources to the non-agricultural department [11,40] and that this labor allocation affects behavior related to cropland allocation (e.g., self-management, transfer, and abandonment) [11].

The results of prior studies indicate that rural labor migration is considered the main factor causing cropland abandonment [17,18,19,21,25]. More specifically, previous studies suggested that there was a positive relationship between off-farm employment and cropland abandonment. However, Chinese law concerning land ownership is unique. According to the law, farmers hold only the right of land management with a time limit, while the collective holds ownership rights without a time limit [41]. Specifically, the law allows the collective to adjust land management rights if two-thirds of the villagers agree to do so, and the law also allows the collective to deprive a villager of the right of land management if the villager abandons the land for two continuous years. Thus, there is much uncertainty about whether farmers will continue to hold the right of land management in the future. Conversely, with the implementation of the “Village Revitalization Strategy”, cropland resources in rural areas may play an essential role in the future, which also means that the relationship between off-farm employment and cropland abandonment in China may be different than in other countries.

Against this backdrop, there may be an inverted U-shaped relationship between off-farm employment and cropland abandonment. When the share of off-farm labor as a percentage of total labor is below a critical value, households do not possess sufficient labor resources to sustain the production of existing land, and peasants who rely on off-farm income may not be able to afford the cost of outsourcing agricultural production. Under these conditions, households may abandon cropland. Prior studies confirm the probability of this scenario occurring in China (e.g., in the provinces of Jiangxi [20] and Jiangsu [22]). Conversely, when the share of off-farm labor compared to total labor is above a critical value, households possess sufficient labor resources to sustain the production of existing land, or peasants who rely on off-farm income may be able to afford the cost of outsourcing agricultural production. Under these conditions, households may not abandon cropland. These scenarios accurately reflect Chinese circumstances. In China, it remains difficult for migrant workers to fully integrate into urban society [42], and land has certain social security functions [43]. As a result, peasants may not relinquish the right of land management [22]. Using their off-farm income to outsource agricultural production, they can avoid abandoning cropland. In fact, the service radius of agricultural machinery has been expanded with the increase in off-farm employment in China [44]. Previous studies may not have paid sufficient attention to the non-linear relationship between off-farm employment and cropland abandonment, and off-farm employment is often simplistically linked to cropland abandonment. This possibility is another motivation for our interest in determining whether there is an inverted U-shaped relationship between off-farm employment and cropland abandonment.

## 3. Materials and Methods

### 3.1. Data Source

The data used in this study come from a household survey that was conducted from June to August 2014 in China, with the China Labor-force Dynamics Survey (CLDS) provided by the Center for Social Science Survey at Sun Yat-sen University in Guangzhou, China. The survey sample used a multistage sampling procedure for observation units. First, 209 sample counties were systematically sampled with a random start based on the sorting of Gross Domestic Product and the scale of labor from 27 provinces in China. Second, 401 sample villages were assigned a probability proportionate to the sample size based on sorting of the migrant population ratio and the scale of labor from the 209 sample counties. Third, 14,214 sample households were systematically sampled with a random start based on the address map of the 401 sample villages. Finally, sample household changes followed sample village changes. The total number of households in the sample was 14,214. During the data analysis, households that always lived in cities were excluded from this study. After cleaning, data from 8031 valid household questionnaires were used for the analysis.

### 3.2. Econometric Method

This study explores the allocation of land by the behavior and area of cropland abandonment. In terms of cropland abandonment behavior, peasants’ decision-making behavior is considered binary (abandonment or non-abandonment). Thus, the values of the dependent variable are binary discrete values (1 for abandonment and 0 for non-abandonment), and this study explores the relationship between off-farm employment and the behavior of cropland abandonment using an IV-Probit model. Regarding the area of cropland abandonment, most peasants do not abandon cropland. Thus, the values of the dependent variable are continuous and include many zeros, and this study explores the relationship between off-farm employment and the area of cropland abandonment with an IV-Tobit model. To check the robustness of the focal variable, this study uses a model that firstly includes only the focal variable and then gradually introduces province dummy controls and other variables. Additionally, this study uses robust standard errors to decrease the effect of heteroscedasticity on the estimated results. The basic models are as follows.

(1) $${Abandon}_{ip}{=\beta }_{0}{+\beta }_{1}{off-farm}_{ip}{+\beta }_{2}{off-farm}_{ip}^{2}{+\gamma X+\delta }_{p}{+\varepsilon }_{ip}$$

(2) $${Area}_{ip}{=\beta }_{0}^{*}{+\beta }_{1}^{*}{off-farm}_{ip}{+\beta }_{2}^{*}{off-farm}_{ip}^{2}{+\gamma }^{*}{X+\delta }_{p}^{*}{+\varepsilon }_{ip}^{*}$$

where the subscripts of i and p respectively represent household and province; Abandon is a dummy variable, in which a value of 1 represents that the household has abandoned cropland and 0 represents otherwise; Area is a continuous variable, which represents the area of cropland abandonment; off-farm is the share of off-farm laborers among the total household laborers; X is a vector of control variables (e.g., age, education, land size and certificate, labor size, assets, etc.); both $\beta_{0}$ and $\beta_{0}^{*}$ are constants; $\beta_{1}$, $\beta_{2}$, $\beta_{1}^{*}$, and $\beta_{2}^{*}$ are estimated parameters; both γ and $\gamma^{*}$ are the vectors of estimated parameters for the control variables; both δ and $\delta^{*}$ are province dummies; both ε and $\varepsilon^{*}$ are random error terms. Additionally, to solve the endogenous problem of off-farm employment, we employ the IV-Probit and IV-Tobit models developed by Newey [45]. The instrument IVoff-farm for off-farm is the average share of off-farm employment for other households in the same village excluding the household under concern (*n* − 1), which is ${IVoff-farm}_{iv}{=(off-farm}_{1}{+off-farm}_{2}{+}_{\cdots }{+off-farm}_{n-1})/(n-1)$.

### 3.3. Variable Design

#### 3.3.1. Dependent Variables

In this study, the dependent variable is cropland abandonment. Cropland abandonment can be separated into behavior and area, i.e., whether rural households abandon cropland and the area of cropland abandonment in rural households.

#### 3.3.2. Focal Variables

In this study, the focal variable is off-farm employment. Specifically, its measure is the share of off-farm labor among total labor. This study defines the labor of off-farm employment following Huang et al. [46], Xu et al. [11], and Ji et al. [12], which matches the following four conditions: (1) workers are aged 16–64, (2) have the ability to work, (3) are not students, and (4) their major source of work is off-farm. Moreover, in order to explore the nonlinear relationship between off-farm employment and cropland abandonment, the square term of off-farm employment is introduced.

#### 3.3.3. Control Variables

To reduce the impact of omitted variables on the estimated results, this study controls characteristics of the household member (e.g., age and education), household (e.g., land area, land right, land rented out, labor size, assets, location, etc.), and province (dummies). The definition and assignment of all the variables are shown in Table 1.

## 4. Results

### 4.1. Spatial Distribution Analysis

**Figure 2:**
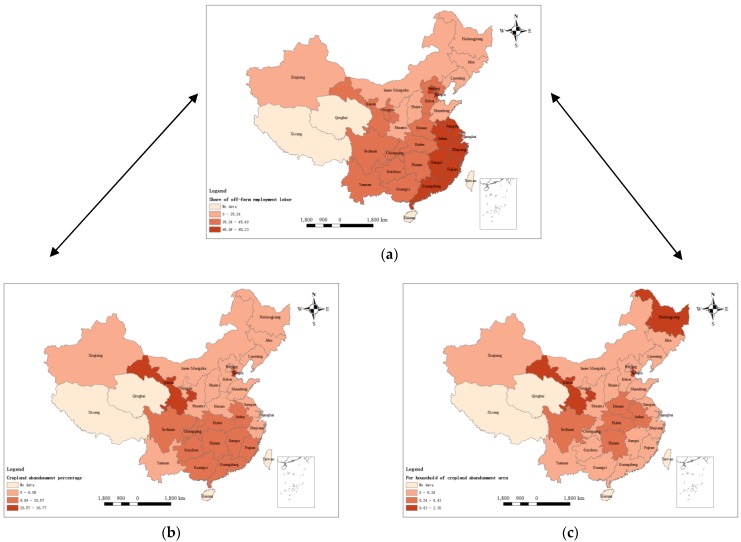
(**a**) Off-farm employment percentage; (**b**) Cropland abandonment percentage; (**c**) Cropland abandonment area.

China is a country with large geographical and spatial differences. Additionally, there are substantial differences in labor migration and cropland abandonment between provinces. This study uses national samples and ArcGIS software (version 10.3.1, Esri Inc., California, CA, USA) to create a spatial distribution map with the province as the basic statistical unit. Off-farm labor, occurrence of cropland abandonment, and cropland abandonment area per household are represented in Figure 2a–c, respectively. As shown in Figure 2a, the districts in which off-farm employment is prevalent exhibit substantial differences in spatial area agglomeration, and these districts are primarily concentrated in China’s economically developed eastern coastal provinces (e.g., Guangdong, Jiangsu, Zhejiang, Fujian). Jiangsu has the highest share of off-farm employment (61.18%), while Xinjiang Province has the lowest share of such employment (1.35%). These agglomeration trends may be related to labor market characteristics, as labor market characteristics will affect farmers’ employment choices [47]. For example, the eastern coastal provinces (such as Guangdong, Jiangsu, Zhejiang, Fujian, etc.) are the major provinces of China’s traditional manufacturing industry, and they attract many laborers from the western region; the central provinces (such as Shanxi, Henan, Hubei, Hunan, etc.) are the major agricultural provinces of China. In recent years, with the transfer of eastern industries to the interior, the proportion of off-farm employment has gradually increased. The western provinces (such as Shaanxi, Inner Mongolia, Xinjiang, etc.) are mostly high-altitude mountainous, which have less-developed economies and lower proportions of off-farm employment. As shown in Figure 2b, the occurrence of cropland abandonment in various provinces of China presents substantial differences in spatial area agglomeration and is primarily concentrated in provinces with complex topography (e.g., Gansu, Ningxia). Gansu exhibits the highest percentage of cropland abandonment (36.77%). As shown in Figure 2c, the cropland abandonment area per household in various provinces of China also presents substantial differences in spatial area agglomeration. Gansu has the largest cropland abandonment area per household (2.19 mu).

Generally, as shown in Figure 2a–c, off-farm employment and the behavior of cropland abandonment display similar trends of spatial area aggregation; i.e., regions with a higher share of off-farm employment are also regions with more cropland abandonment. However, only some of the regions with higher shares of off-farm employment are also regions with more cropland abandonment. From the perspective of spatial characteristics analysis, it remains unclear whether off-farm employment is a key driving determinant of cropland abandonment. Therefore, we adopt econometric models to verify whether various correlation coefficients are significant.

### 4.2. Empirical Analysis

#### 4.2.1. Off-Farm Employment Impacts Behavior of Cropland Abandonment (IV-Probit)

The estimated results for the impact of off-farm employment on cropland abandonment are shown in Table 2. As previously indicated, the IV-Probit model is employed. As shown in Table 2, in Models 1–3, the dependent variable is whether rural households abandon cropland. The independent variables cumulatively include off-farm employment and its square, province dummies, and other control variables. Model 4 represents the marginal results based on Model 3. The endogenous Wald *χ*^2^ values of all models are significant at the 1% level. Thus, the core independent variable of the model is an endogenous variable, and it is appropriate to estimate the model with an IV-Probit model.

As shown in Table 2, off-farm employment and its square are always significant at the level of 1% and have opposite signs in all models (i.e., off-farm employment and its square have positive and negative signs, respectively), which indicate that there is a non-linear and inverted U-shaped relationship between off-farm employment and cropland abandonment. Based on the marginal estimated results of Model 4, there is a turning point when the share of off-farm employment labor relative to total labor accumulates to the level of 46.00% (−0.0184/2 × (−0.0002)). In addition, control variables have econometric meanings in Model 4, as follows. (1) The householder’s age and its square are significant at the level of 1%, and the square has a positive sign. Thus, there is a U-shaped relationship between a householder’s age and cropland abandonment. (2) Land area significantly and positively affects cropland abandonment. More precisely, when the land controlled by a household increases by 1 mu, the possibility of cropland abandonment increases by 0.0006%. (3) Land rent-out significantly and negatively affects cropland abandonment. More precisely, if households participate in land rent-out, the possibility of cropland abandonment decreases by 0.0750%. (4) Labor size significantly and negatively affects cropland abandonment. More precisely, when every rural household adds one laborer, the possibility of cropland abandonment decreases by 0.0429%. (5) Fixed assets and agricultural assets significantly and negatively affect cropland abandonment. (6) If rural households are located in hilly or mountainous areas, they are more likely to abandon cropland. 

#### 4.2.2. Off-Farm Employment Effects Area of Cropland Abandonment (IV-Tobit)

The estimated results for the impact of off-farm employment on cropland abandonment area are shown in Table 3. As previously indicated, the IV-Tobit model is employed. As shown in Table 3, in Models (1)–(3), the dependent variable is cropland abandonment area of the rural household. The independent variables cumulatively include off-farm employment and its square, province dummies, and other control variables; Model (4) represents the marginal results based on Model (3). The endogenous Wald *χ*^2^ values of all the models are significant at the 1% level. Thus, the core independent variable of the model is an endogenous variable, and it is appropriate to estimate the model with an IV-Tobit model.

As shown in Table 3, off-farm employment and its square are always significant at the level of 1% and have opposite signs in all models (i.e., off-farm employment and its square have positive and negative signs, respectively), which indicate that there is a non-linear and inverted U-shaped relationship between off-farm employment and the area of cropland abandonment. Based on the marginal estimated results of Model (4), there is a turning point when the share of off-farm labor relative to total labor reaches a level of 44.50% (−0.0178/2 × (−0.0002)). In addition, control variables have econometric meanings in Model (4), as follows: (1) The householder’s age and its square are significant at the level of 1%, and the square has a positive sign. Thus, there is a U-shaped relationship between householder age and abandonment area; (2) Land area significantly and positively affects the abandonment area. More precisely, when the land controlled by a household increases by 1 mu, the area of cropland abandonment increases by 0.0019 mu; (3) Land rent-out significantly and negatively affects abandonment area. More precisely, if households participate in land rent-out, the area of cropland abandonment decreases by 0.0707 mu; (4) labor size significantly and negatively affects the area. More precisely, when every household increases by one more laborer, the area of cropland abandonment decreases by 0.0390 mu; (5) both fixed assets and agricultural assets significantly and negatively affect cropland abandonment behavior.

#### 4.2.3. Labor Off-Farm Employment Does Not Inevitably Result in Cropland Abandonment

**Figure 3:**
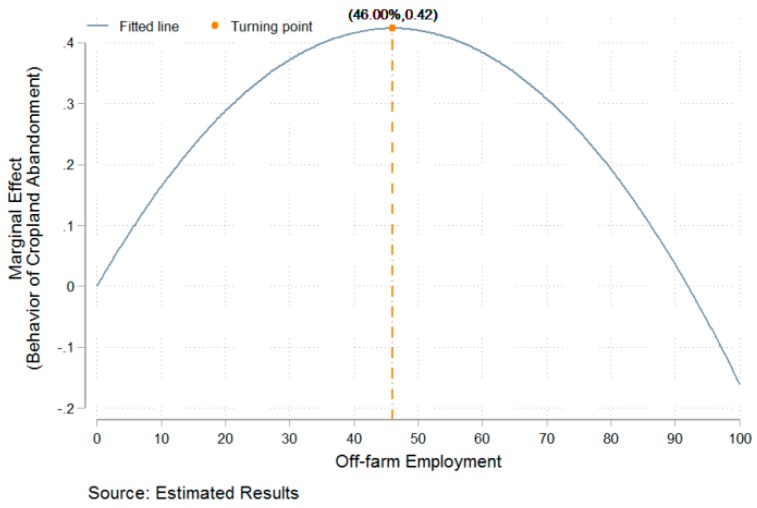
The function of marginal effect of off-farm employment on behavior.

**Figure 4:**
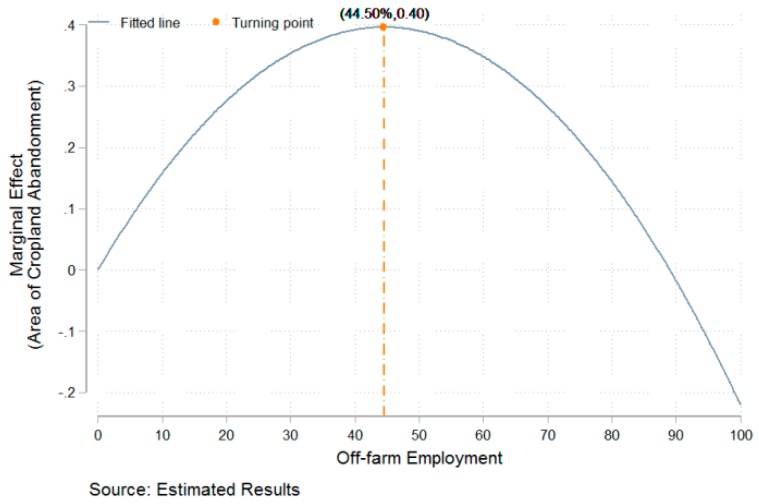
The function of marginal effect of off-farm employment on area.

The described empirical results indicate that there is an inverted U-shaped relationship between off-farm employment and cropland abandonment. Based on the estimated results of Model 4 in Table 2, a map was created that describes the marginal effect of off-farm employment on cropland abandonment (Figure 3). According to the estimated result of Model 4 in Table 3, a map was created that describes the marginal effect of off-farm employment on the area of cropland abandonment (Figure 4). As shown in Figure 3, the critical value is 46%. In other words, when the share of off-farm labor as a function of total labor is lower than 46%, the possibility of households abandoning cropland increases as the off-farm labor share increases; in contrast, when the share of off-farm labor among total labor is higher than 46%, the possibility of households abandoning cropland decreases as the off-farm employment share increases. As shown in Figure 4, the critical value is 44.50% for cropland abandonment area. When the share of off-farm labor among total labor is lower than 44.50%, the area of household abandoned cropland increases as the off-farm labor share increases; in contrast, when the share of off-farm labor among total labor is higher than 44.50%, the area of household abandoned cropland decreases as the off-share labor share increases. In sum, there is an inverted U-shaped relationship between off-farm employment and cropland abandonment; i.e., off-farm employment does not inevitably result in cropland abandonment. 

#### 4.2.4. Robustness Checks

To account for the double-way causation, this study employs an IV-Probit model and an IV-Tobit model to investigate the relationship between off-farm employment and cropland abandonment. The identification strategy is to add control variables step by step, which helps overcome regression bias resulting from omitted variables. Unfortunately, nonrandom selection and measurement error can harm estimate robustness. To ensure robustness, the two following strategies are adopted.

The first strategy is to eliminate the impact of nonrandom selection on the estimated results by employing a subsample of seven provinces randomly extracted from the primary sample. The estimated results are shown in Models 1 and 3 of Table 4. The second strategy is to eliminate the impact of measurement errors on the estimated results, which constructs a new focal variable defined as the share of off-farm income of total income. The estimated results are shown in Models 2 and 4 of Table 4.

As shown in Table 4, regarding cropland abandonment, the estimated results change little, regardless of whether regression by subsample (i.e., the first strategy) or a new focal variable (i.e., the second strategy) is used. The only difference is the value of the coefficient. Similarly, regarding the area of cropland abandonment, whether regressions by subsample (i.e., the first strategy) or a new focal variable (i.e., the second strategy) is used, the estimated results change little. The only difference is the value of the coefficient. Generally, the results shown in Table 4 reveal that, regardless of whether subsamples or new focal variables are used, there is a non-linear, inverted U-shaped relationship between off-farm employment and cropland abandonment after the endogeneity problem is resolved.

## 5. Discussion

Based on a large data sample of peasants from 27 Chinese provinces, this study seeks to answer two key scientific questions regarding the spatial distribution of and the quantitative relation between off-farm labor and cropland abandonment. Compared to prior studies, the contributions of this study are as follows. (1) From a spatial perspective, we reveal the distribution of off-farm employment and cropland abandonment, whereby the province is regarded as the basic statistical unit. (2) From a quantitative perspective, we investigate the non-linear relationship between off-farm employment and cropland abandonment. In addition, the sample of this study analyzes data for 27 provinces in China (approximately 87% of the provinces of mainland China), including data for 37,059 peasants from 8031 peasant households (approximately 0.005% of the total peasant population of mainland China). Compared to other studies with small samples, the results of this study may be more representative of the actual circumstances in China, and the conclusions are more universal. The study results can also serve as more useful references in the rational allocation of labor and cropland resources and the formulation of relevant policies, such as those regarding food security. 

Cropland abandonment has become a common phenomenon across the globe [48,49]. Research on cropland abandonment has mainly focused on developed countries such as European countries and the United States [49]. Those studies suggested that off-farm employment can lead to cropland abandonment from the perspective of qualitative analysis [17,21] or macroscopic quantitative analysis [18,19,25,50,51]. In contrast, this study explores the marginal impact of off-farm employment on cropland abandonment from the perspective of microscopic quantitative analysis. Interestingly, this study indicates that there is a non-linear and inverted U-shaped relationship between off-farm employment and the behavior/area of cropland abandonment, the turning point for which is 46.00% or 44.50% of off-farm labor among total labor. In other words, this study considers that off-farm labor does not inevitably result in cropland abandonment. A possible explanation for these differences is as follows. On the one hand, the large number of rural laborers employed in off-farm sectors causes a shortage of agricultural labor [40], and rural regions lack a market for land transfers [14]. Households do not possess sufficient labor resources to sustain the production of existing land, and peasants who rely on off-farm income may not be able to afford the cost of outsourcing agricultural production. Thus, households may abandon cropland. On the other hand, there is much uncertainty about whether farmers in China will continue to hold the right of land management. In addition, it remains difficult for migrant workers to fully integrate into urban society [42], and land owned by peasants can provide them a degree of social security [43]. Thus, peasants may not relinquish the right of land management [22]. Peasants who rely on off-farm income may outsource agricultural production to avoid abandoning cropland. 

This study has several deficiencies, which can be addressed in future research. (1) Cross-sectional data are employed to discuss the relationship between off-farm employment and cropland abandonment. Because the relationship between off-farm employment and cropland abandonment is dynamic, future research can employ panel data to analyze that relationship. (2) In China, the right of land ownership belongs to the collective. The peasant household only holds the right of land management, while in certain countries, the rights of land ownership and management vest in the same individual (the collective or the private). Future research can verify whether the conclusion of this study is applicable in those countries.

## 6. Conclusions

This study uses the data on 8031 peasant households collected by the Center for Social Science Survey of Sun Yat-sen University in 2014. Based on these data, the study investigates the relationship between off-farm employment and cropland abandonment through spatial and empirical analysis. The results are as follows:

(1) The spatial distribution of off-farm employment and cropland abandonment differs among regions. A region with a higher ratio of off-farm employment shows more cropland abandonment but a lower average area of cropland abandonment.
(2) Off-farm labor has a significant and positive correlation with cropland abandonment. However, its square shows a significant and negative correlation with cropland abandonment; i.e., there is an inverted U-shaped relationship between off-farm employment and cropland abandonment, with the turning point occurring at 46.00% off-farm employment.
(3) Off-farm employment has a significant and positive correlation with the area of cropland abandonment. However, its square has a significant and negative correlation with the area; i.e., there is an inverted U-shaped relationship between off-farm employment and area, with the turning point occurring at 44.50% off-farm employment.

Our results have several policy implications. For example, the inverted U-shaped relationship we establish implies that policymakers should focus on improving the income of rural migrants, enhancing rural infrastructure, and guiding peasants to use off-farm income to improve agricultural production conditions. Additionally, land rent-out helps reduce cropland abandonment. Thus, to decrease land misallocation, policymakers should focus on building a mature land transfer market, which would also help improve agricultural productivity to ensure food security.

## Figures and Tables

**Table 1:** The definition and data description of the variables in the model.

Variables	Definition and Assignment	Mean	S.D. ^c^
Dependent variables			
Abandonment	Whether rural households abandon cropland (0 = no; 1 = yes)	0.12	0.32
Abandonment size	The area of cropland abandonment in rural households (mu ^a^)	0.33	1.65
Focal variable			
Off-farm employment	The share of off-farm employment laborers in total laborers (%)	0.40	0.39
Householder variables			
Age	Age of household head in years (year)	53.80	13.20
Education	Whether householder has received a high school diploma or above (0 = no; 1 = yes)	0.12	0.32
Household variables			
Land area	Managing land area of rural households (mu ^a^)	5.72	9.60
Land right	Whether rural households get the land confirmation certificate (0 = no; 1 = yes)	0.41	0.49
Land rent-out	Whether there is rent-out land in rural households (0 = no; 1 = yes)	0.69	0.46
Old	The number of people over 64 years old engaged in agricultural production (number)	0.15	0.46
Labor size	Total labor force of rural households (number)	2.74	1.60
Distance	Distance from households to the nearest business center (km)	7.12	9.18
Agricultural assets	Per capita of current market value of all the agricultural assets that a household possesses (10^4^ Yuan ^b^/person)	0.08	0.53
Fixed assets	Per capita of current market value of all the fixed assets that a household possesses (10^4^ Yuan ^b^/person)	4.32	16.75
Plain	Whether households are located on a plain (0 = no; 1 = yes)	0.41	0.49
Hill	Whether households are located on a hill (0 = no; 1 = yes)	0.34	0.47
Mountain	Whether households are located on a mountain (0 = no; 1 = yes)	0.25	0.43

^a^ 1 mu ≈ 667 m^2^ or 0.067 ha. ^b^ During the study period, 1 USD was equal to 6.12 Chinese Yuan. ^c^ standard deviation.

**Table 2:** The estimated results of IV-Probit model and its marginal effect ^a^.

Variables	Model (1)	Model (2)	Model (3)	Model (4)
Off-farm employment	0.0646 ***	0.0637 ***	0.0758 ***	0.0184 ***
	(0.0040)	(0.0076)	(0.0056)	(0.0016)
Off-farm employment^2^	−0.0007 ***	−0.0007 ***	−0.0008 ***	−0.0002 ***
	(0.0000)	(0.0001)	(0.0001)	(0.0000)
Age			−0.0325 ***	−0.0079 ***
			(0.0068)	(0.0017)
Age^2^			0.0004 ***	0.0001 ***
			(0.0001)	(0.0000)
Education			0.0293	0.0071
			(0.0496)	(0.0120)
Land area			0.0026 *	0.0006 *
			(0.0014)	(0.0003)
Land right			−0.0268	−0.0065
			(0.0333)	(0.0081)
Land rent-out			−0.3089 ***	−0.0750 ***
			(0.0475)	(0.0106)
Old			−0.0085	−0.0021
			(0.0351)	(0.0085)
Labor size			−0.1769 ***	−0.0429 ***
			(0.0198)	(0.0053)
Distance			0.0013	0.0003
			(0.0017)	(0.0004)
Fixed assets			−0.0297 *	−0.0072 *
			(0.0173)	(0.0042)
Agricultural assets			−0.2034 *	−0.0494 *
			(0.1058)	(0.0256)
Hill			0.0750 *	0.0182 *
			(0.0453)	(0.0109)
Mountain			0.1260 **	0.0306 **
			(0.0503)	(0.0121)
Constant	−1.2225 ***	−1.3527 ***	−0.1847	
	(0.0395)	(0.2190)	(0.2646)	
Province dummies	No	Yes	Yes	Yes
Endogenous Wald *χ*^2^	436.64 ***	183.69 ***	676.25 ***	676.25 ***
Observations	8031	8031	8031	8031

^a^ Robust standard errors in parentheses; * Significant at α = 0.10; ** significant at α = 0.05; *** significant at α = 0.01.

**Table 3:** The estimated result of IV-Tobit model and its marginal effect ^a^.

Variables	Model (1)	Model (2)	Model (3)	Model (4)
Off-farm employment	0.7209 ***	0.5467 ***	0.7129 ***	0.0178 ***
	(0.0590)	(0.0738)	(0.0845)	(0.0013)
Off-farm employment^2^	−0.0080 ***	−0.0059 ***	−0.0076 ***	−0.0002 ***
	(0.0007)	(0.0009)	(0.0010)	(0.0000)
Age			−0.3035 ***	−0.0076 ***
			(0.0704)	(0.0017)
Age^2^			0.0034 ***	0.0001 ***
			(0.0006)	(0.0000)
Education			0.3087	0.0077
			(0.5574)	(0.0138)
Land area			0.0758 ***	0.0019 ***
			(0.0218)	(0.0005)
Land right			−0.4077	−0.0102
			(0.3233)	(0.0080)
Land rent-out			−2.8267 ***	−0.0707 ***
			(0.3875)	(0.0095)
Old			−0.0859	−0.0021
			(0.3382)	(0.0085)
Labor size			−1.5602 ***	−0.0390 ***
			(0.2132)	(0.0040)
Distance			0.0143	0.0004
			(0.0166)	(0.0004)
Fixed assets			−0.3719 **	−0.0093 **
			(0.1718)	(0.0043)
Agricultural assets			−3.0541 **	−0.0764 ***
			(1.2333)	(0.0291)
Hill			0.3120	0.0078
			(0.4327)	(0.0107)
Mountain			0.2234	0.0056
			(0.4777)	(0.0119)
Constant	−12.4638 ***	−12.3135 ***	−3.1040	
	(0.7721)	(1.8446)	(2.8156)	
Province dummies	No	Yes	Yes	Yes
Endogenous Wald *χ*^2^	856.33 ***	276.69 ***	697.48 ***	697.48 ***
Observations	8031	8031	8031	8031

^a^ Robust standard errors in parentheses; * Significant at α = 0.10; ** significant at α = 0.05; *** significant at α = 0.01.

**Table 4:** The estimated results of robustness checks ^a^.

Variables	Behavior of Cropland Abandonment		Area of Cropland Abandonment	
	Model (1)	Model (2)	Model (3)	Model (4)
Off-farm employment	0.0788 ***	0.0768 ***	0.8636 ***	1.3886 ***
	(0.0032)	(0.0017)	(0.1036)	(0.1349)
Off-farm employment^2^	−0.0008 ***	−0.0008 ***	−0.0093 ***	−0.0144 ***
	(0.0000)	(0.0000)	(0.0011)	(0.0014)
Control variables	Yes	Yes	Yes	Yes
Province dummies	Yes	Yes	Yes	Yes
Endogenous Wald *χ*^2^	785.62 ***	386.41 ***	1300.00 ***	179.62 ***
Observations	3266	7996	3266	7996

^a^ Robust standard errors in parentheses; *** significant at α = 0.01.
